# The impact of insecticide-treated school uniforms on dengue infections in school-aged children: study protocol for a randomised controlled trial in Thailand

**DOI:** 10.1186/1745-6215-13-212

**Published:** 2012-11-15

**Authors:** Annelies Wilder-Smith, Peter Byass, Phanthip Olanratmanee, Pongsri Maskhao, Luechai Sringernyuang, James G Logan, Steve W Lindsay, Sarah Banks, Duane Gubler, Valérie R Louis, Yesim Tozan, Pattamaporn Kittayapong

**Affiliations:** 1 Centre for Global Health Research, Department of Public Health and Clinical Medicine, Umeå University, Umeå, Sweden; 2 Institute of Public Health, Heidelberg University Medical School, Heidelberg, Germany; 3 Center of Excellence for Vectors and Vector-Borne Diseases, Faculty of Science, Mahidol University at Salaya, Nakhon Phatom, Thailand; 4 Faculty of Humanities and Social Sciences, Rajabhat Rajanagarindra University, Chachoengsao, Thailand; 5 Faculty of Social Sciences and Humanities, Mahidol University at Salaya, Nakhon Pathom, Thailand; 6 Department of Disease Control, London School of Hygiene and Tropical Medicine, London, UK; 7 Emerging Infectious Diseases Program, Duke-NUS, Singapore, Singapore; 8 Department of International Health, Boston University School of Public Health, Boston, MA, USA

**Keywords:** Dengue, Insecticide-treated clothes, School children, School uniforms, Randomised control trial, Cost effectiveness

## Abstract

**Background:**

There is an urgent need to protect children against dengue since this age group is particularly sensitive to the disease. Since dengue vectors are active mainly during the day, a potential target for control should be schools where children spend a considerable amount of their day. School uniforms are the cultural norm in most developing countries, worn throughout the day. We hypothesise that insecticide-treated school uniforms will reduce the incidence of dengue infection in school-aged children. Our objective is to determine the impact of impregnated school uniforms on dengue incidence.

**Methods:**

A randomised controlled trial will be conducted in eastern Thailand in a group of schools with approximately 2,000 students aged 7–18 years. Pre-fabricated school uniforms will be commercially treated to ensure consistent, high-quality insecticide impregnation with permethrin. A double-blind, randomised, crossover trial at the school level will cover two dengue transmission seasons.

**Discussion:**

Practical issues and plans concerning intervention implementation, evaluation, analysing and interpreting the data, and possible policy implications arising from the trial are discussed.

**Trial registration:**

clinicaltrial.gov. Registration number: NCT01563640

## Background

Dengue viruses are among the most geographically widespread of the arboviruses and are found in tropical and subtropical areas, where 2.5–3 billion people are at risk of infection [1]. Each year an estimated 230 million dengue infections occur; these infections result in over 2 million cases of dengue haemorrhagic fever (DHF) and about 20,000 deaths [1-3]. Globally, the number of disability-adjusted life years (DALYs) lost to dengue is estimated to range between 528 and 621 per million population, a significantly high number that warrants enhanced control strategies [4]. The World Health Organisation (WHO) states that the Southeast Asian and Western Pacific regions bear nearly 75% of the current global disease burden of dengue [4]. Thailand is one of the countries with the highest dengue incidence [5].

Children carry the main burden of morbidity and mortality for dengue with a higher rate of complications, including DHF and shock syndromes, than in adults [6]. Infection in children causes disruptions in schooling and parental wage earning, which in turn have major direct effects on nutrition and overall family health. It is estimated that dengue contributes 465·3 DALYs per million population per year among school-aged children in northern Thailand, which accounts for 15% of all DALYs lost from febrile illness in this age group [7].

Past dengue control efforts have generally focussed on vector control but have been largely ineffective [4]. There is an urgent need for integrated and complementary population-based strategies to protect vulnerable children. The dengue vectors, *Aedes aegypti* and *Ae. albopictus*, are active mainly during the day when children are at school. Therefore, schools are potentially a key target for control. School uniforms are a cultural norm in most developing countries and are worn throughout the day on an almost daily basis.

Insecticide-treated fabrics have emerged as a key component of malaria disease control efforts following the widespread increased use of long-lasting insecticidal nets (LLINs) in recent years [8]. However, LLINs are likely to be relatively ineffective for dengue control since transmission occurs during the day when people are outside the protection of their nets. There have been a considerable number of strategies that have applied insecticide to personal clothing, but the application has been limited to military and recreational markets and has not reached community-based protection [9,10].

The most common insecticide applied to clothing is permethrin, a pyrethroid, which has an excellent safety record [11,12]. Permethrin is registered with the US Environmental Protection Agency (EPA) and approved by WHO for use in fabrics and clothing. Although the insecticide will transfer from the fabric to the skin, there is little evidence of adverse effects, and when used at appropriate concentrations, insecticide-treated clothing is deemed safe [13].

Commercially available fabrics made from a proprietary permethrin formula bound tightly to fabric fibres result in an effective, odourless protection against many biting arthropods including *Anopheles*, *Culex* and *Aedes* mosquitoes [14-16], ticks [17,18], chigger mites [19,20], and body lice [15]. Studies have shown that wearing permethrin-treated clothing led to reduced biting rates of *Aedes* mosquitoes by >90% in the areas covered by the clothing [21]. Although permethrin-treated clothing is used predominantly in recreational markets and the military, some studies have demonstrated that it can impact disease transmission [22]. For example, a randomised controlled trial of Colombian soldiers found that wearing permethrin-impregnated uniforms significantly reduced the incidence of malaria and cutaneous leishmaniasis by 75% compared with wearing non-impregnated uniforms [10]. The potential of using impregnated clothing and bedding has been shown in Pakistan [23] and Kenya [24] for malaria control. Tests have also shown that factory-based dipping methods of impregnating clothing can retain an effective repellence and knockout effect after 70 washings [25].

We aim to test our hypothesis [26] that insecticide-treated school uniforms will reduce the incidence of dengue in school-aged children. We will conduct a community-based randomised controlled trial in Thailand. This trial is conducted by partners of research area 2 in the “DengueTools” consortium [27].

### Study objectives

#### Primary objective

The primary objective is to assess the impact of impregnated pre-fabricated school uniforms on laboratory-confirmed dengue incidence in school-aged children.

### Secondary objectives

#### Clinical

To assess the impact of impregnated school uniforms on the number of febrile episodes during the study period (compared to the control group)

To assess the impact of impregnated school uniforms on school absenteeism (number of days lost because of febrile illness)

To measure the protective herd effect (halo effect) on dengue incidence in children without impregnated clothes

To assess the safety of impregnated school uniforms in children

#### Entomological and ecological

To investigate the effect of impregnated school uniforms on vector abundance in and around schools

To determine whether impregnated clothing affects behaviours associated with adult mosquito feeding and indoor resting

To determine the environmental and socioeconomic risk factors associated with dengue infections

#### Economic analysis

To estimate the costs of dengue infection in school-aged children

To estimate the costs and cost-effectiveness of impregnated school uniforms for the prevention of dengue in school-aged children

#### Social science

To assess the behaviour of Thai school-aged children with regard to wearing the school uniform and washing practices of school uniforms

To assess the perception of dengue severity in the community (school teachers and parents)

To assess the acceptability of impregnated school uniforms in children

To assess community acceptance of impregnated school uniforms and the trial

## Methods/Design

### Study area and participant eligibility

The study area is located about 150 km east of Bangkok at transmission foci in Plaeng Yao District, Chachoengsao Province, eastern Thailand. Our Thai research group has already established excellent relationships with local schools because of a decade of research in this area [28-30]. The climate consists of a rainy season from May to October followed by a long dry season. The region covers an area of 237 km^2^ and a population of 36,607 persons (census 2005; http://en.wikipedia.org/wiki/Plaeng_Yao_District). There are 24 primary and secondary schools in this area, of which we will select 10 schools that have between 100–500 students who are willing to participate. All children aged 7 to 18 years old will be eligible. In order for the results from this study to be as generalisable as possible, with the exception of children with eczema or other severe underlying skin diseases, no exclusions will be made on the basis of gender, ethnic group, medical condition or physical health. Inclusion will require parents/caretakers to give informed written consent; in the case of children older than 12 years, assent will also be required. Subjects and households will be free to withdraw from participating in the study at any time without giving a reason. To ensure a high compliance rate among children in participating schools, awareness campaigns in the community and in the schools will be held. Information sessions with the school leadership, school teachers and parents will be conducted between October 2011 and May 2012 before the start of trial.

Study subjects will be enrolled between January and June 2012. The intervention/control will commence in mid-May to June 2012, at the start of the Thai school year.

### Intervention

Routinely used school uniforms will be collected from all consenting children and treated with a long-lasting permethrin formulation, using a factory proprietary method. The process involves coating the uniforms in a proprietary formula containing 0.52% permethrin (Insect Shield USEPA2009). The technology claims to provide 80–96.7% knockdown after 70 washes [25]. Enrolled children will receive either permethrin impregnated or untreated school uniforms. For schools assigned to the control group, uniforms will be collected and washed using the same methodology as those used in the treatment group, but without the impregnation process with permethrin.

School uniforms vary in form, colour and size. School uniforms include the regular school uniform, scouts uniform, sports uniform and cultural uniform. Most of the typical uniforms are short-sleeved and only cover the legs down to the knees (shorts or skirts). The variation between the schools is minimal for the uniforms, except for the cultural uniform.

### Design

A cluster-randomised, double-blinded, crossover design will be implemented. Randomisation will be by school. An individual participation rate of at least 90% within each school is anticipated. The schools will be randomised into two equal groups, with each school receiving impregnated uniforms in either the first (2012–13) or second (2013–14) annual transmission season covered by the trial.

### Randomisation and blinding

There will be double-blinding: neither the children nor the investigators will know the allocation. School uniforms from Thailand will be sent to the InsectShield factory: one group will undergo washing and treatment with permethrin. The control group will only undergo washing. Both the impregnated and washed uniforms will be identified with labels indicating the child’s name, school and year, but there will be no identifiable indication as to which uniforms are actually impregnated.

All participating schools will be stratified into two by enrolment size, above and below median. Randomisation to two groups (intervention followed by control, control followed by intervention) will be carried out in Sweden. This information will be kept confidential between one epidemiologist and the impregnation facility.

### Sample size

Sample size for the trial will take into account likely levels of dengue incidence, as well as the considerable wide-area variation in transmission from season to season, and local area (i.e., per school) variation [31]. Such large potential variations are unpredictable and difficult to quantify. The advantage of the crossover school-randomised design – which can be interpreted as a cluster-randomised design in relation to individuals – is that if there are particular factors driving local area variation from year to year (such as mosquito breeding sites close to schools), those sources of variation will, to some extent, be controlled by the crossover design. This design efficiency is a considerable advantage, notwithstanding the relatively large design effect that needs to be applied to the overall sample size in recognition of the cluster randomisation and the considerable uncertainties around wide-area incidence rates from year to year.

In view of the probable large size of these variations and their inherent uncertainty, a design factor of 3 is used.

A design factor greater than unity is justified because of the school clustering effect. Unfortunately, because of the paucity of knowledge of dengue epidemiology, both in terms of year-on-year variation and its determinants in Thailand in general, and compounded by possible local variations in dengue that might occur between school locations, it is impossible to make detailed calculations of the required design effect. The choice of 3 as the design effect was therefore adopted as a conservative estimate in the absence of being able to make any better estimate.

The basic assumptions underlying the sample size for the trial are an incidence rate (symptomatic plus asymptomatic) of an average of 5% during a transmission season [32], i.e. 10% over two seasons; an aim of halving incidence by using impregnated uniforms (since any smaller effect would not be of policy interest) and a dropout rate (children leaving the school, etc.) of 20%.

According to Schouten and Kester [33], the overall sample size for a non-clustered crossover design can be calculated as

$$2n=\frac{{\left[z_{1}-\alpha /2\sqrt{\pi_{A}\left(1-\pi_{B}\right)+\pi_{B}\left(1-\pi_{A}\right)}+z_{1}-\beta \sqrt{\pi_{A}\left(1-\pi_{A}\right)+\pi_{B}\left(1-\pi_{B}\right)}\right]}^{2}}{{\left(\pi_{A}-\pi_{B}\right)}^{2}}$$

For comparing a 10% incidence with 5%, this amounts to an overall sample size of 508 (90% power, *p* = 0.05). Increasing this figure by 20% to allow for dropout takes this to 610, and allowing for 10% of children to become infected and take no further part in the trial takes this to 670. A design effect of 3 then takes the total sample size to 2,012 (i.e., 1,006 in each study arm).

### Determining new dengue infections during the study period

Blood samples will be collected via venipuncture (2 ml) or finger prick (<0.2ml) from all study subjects at the beginning of the school term (June 2012) and at the end of the first study period (November 2012). Dengue IgG ELISA (with titres) will be measured at the Center of Excellence for Vectors and Vector-Borne Diseases (CVVD), Faculty of Science, Mahidol University at Salaya. The paired serum samples from each volunteer will be screened quantitatively for anti-dengue IgG antibodies by ELISA using a standardised protocol and reagents.

Passive and active surveillance for dengue and febrile episodes will be maintained throughout the study period (school term).

The parents of any child treated for a febrile episode during the study period will be asked to bring their child to the nearest health facility if the child does not show improvement within 48 h. When such study subjects report to a health facility, government nurses or doctors will treat the child following national guidelines and inform the study nurses or local public health officers stationed in the study area. Blood samples for dengue IgM and IgG and dengue PCR will be taken if there is a fever > 37.5°C. In case the health centre has no facilities for taking blood, the child will be advised to go to a centre with such facilities. The blood samples will be sent to a specified laboratory centre in Bangkok.

To enhance passive surveillance, we will also undertake active surveillance of febrile episodes. During the school term, field assistants will daily check from the school records for children who have been reported as absent for 2 days. The caretakers of those children will be contacted to find out about the reason for absenteeism. For those children who are reported to be absent because of a febrile illness, a blood sample will be taken within 4 weeks after onset of illness to check for dengue IgM. Furthermore, any laboratory data and clinical information will be collected directly from the health centre in case the child has gone to such a health centre or hospital.

### Laboratory definition of dengue infection

#### Evidence of recent dengue virus infection

This is defined as detectable IgM dengue antibody, or positive dengue PCR and/or NS1 Antigen, as sampled from the passive and active surveillance.

#### Dengue virus infection during the study period

This is defined by quantitative dengue IgG ELISA, as a fourfold increase in antibody titres against any dengue virus serotype between the baseline and end of study specimens in paired sera.

### Entomological collections

Mosquito abundance in ten study schools will be measured. One BG sentinel trap per school will be randomly placed indoors at the beginning of the school day (8.30 am) and the mosquitoes will be collected at the end of the school period (3.30 pm). In addition, mosquitoes in each classroom will be collected by portable vacuum aspirators operated for 5 min per classroom. In order to determine species composition of mosquitoes around the school areas, ten ovitraps per school will be randomly placed indoors and outdoors around the school areas and will be left for 7 consecutive days before collection. Eggs collected from ovitraps will be reared to adults and the species will then be identified in the laboratory. These measurements will be taken at baseline and then every 4 weeks to compare entomological changes between the intervention and control schools.

To determine whether there is loss of efficacy against mosquitoes, throughout the entire study period five items of clothing from the ten schools will be examined monthly. This will be done using standardised WHOPES-recommended cone tests.

### Economic analysis

Using a cost-of-illness approach [34], we will examine the economic impact of dengue infection in school-aged children at the household level. While considering the direct costs and indirect costs on households resulting from an illness episode, our analysis will exclude psychosocial costs that are difficult to estimate. A patient questionnaire will be administered to mothers/caretakers of children who have had a febrile illness episode. The questionnaire will document demographic and socioeconomic information of households, characteristics of the child’s illness episode (average duration of fever and illness and symptoms), health-seeking behaviour (type and setting of care received), household spending (direct medical costs and non-medical costs), work and school absenteeism (number of days lost for paid work and from school), informal care costs (hours of patient care provided by household members), and household income lost because of child’s illness episode.

The economic evaluation will include the cost-effectiveness analysis of insecticide-treated uniforms for the prevention of dengue infection based on the intervention efficacy and cost data derived from the trial. The cost-effectiveness analysis will follow standard guidelines of economic analyses and will be conducted from the societal perspective [35]. The intervention costs will be identified, measured and valued alongside the trial. Healthcare resource use data will come from the cost-of-illness study described above. Unit costs for ambulatory and inpatient care will be derived from national published data. Effectiveness will be measured in terms of dengue cases and DALYs averted because of the intervention. To assess the impact of uncertainty in key input variables on the cost-effectiveness results, we will undertake probabilistic uncertainty analysis using Monte Carlo simulations.

### Social science

Quantitative questionnaires in randomly selected students from four schools will be used to determine the main activities of children before and after school in terms of outdoor versus indoor behaviour, types of activities undertaken before and after school, and whether the school uniform is worn after school and for how long, including how the school uniforms are treated (washed and dried) between use. Furthermore, qualitative studies will be used in a small number of randomly selected students to evaluate the washing habits at home. Home visits will be done and key informant interviews conducted with those household members in charge of laundry washing. The purpose is to describe the main methods of washing, drying and ironing.

Movement of children will be assessed using brief questionnaires to estimate the time at risk and usage of the allocated uniforms.

### Risk mapping

Spatial clustering analysis of dengue infections will be carried out to determine transmission foci using geographical information systems (GIS). Locations of schools and study participant home residences will be mapped. Dengue cases will be reported geographically to detect potential hot spots or spatial transmission patterns.

### Analytical plan

The primary endpoint is a comparison of the incidence rates of laboratory-confirmed dengue infections per person-time exposed up to failure (i.e. dengue infection) or censoring (i.e. moving away) between the intervention and control groups.

### Safety considerations

There are no apparent risks to the safety of individuals or communities in this study. Due to its low toxicity and long-lasting efficacy, permethrin is one of the most commonly used insecticides in impregnated clothing. Low irritancy and odour also add to the popularity of permethrin-impregnated clothing [19]. Permethrin-treated uniforms have been fully evaluated by multiple governments for use in military uniforms, for example in the US, Germany and France [21,36]. Even though there is some absorption of the permethrin through the skin, the amount absorbed is well below the acceptable daily intake, and health side effects are not expected [13,37,38]. Insect Shield has met all health requirements for approval by the United States Environmental Protection Agency (USEPA) and has maintained these health standards since 2006 (Insect Shield USEPA, 2009). They are approved for vector control, and the products will be used in compliance with their recommended use and guidelines.

Teachers will document any skin irritations initially every 2 weeks, then every 4 weeks during the study period. Should skin irritations occur, the students will be sent to the study medical clinics for further documentation by trained personnel. In the case that a participant develops a serious adverse event (SAE) during the course of the study, this will be captured in the case report form. In case of an SAE, the study physician will record and manage the SAE in accordance with Good Clinical Practice (GCP). Excessive clustering of SAE by school will be reported to the study’s Data Monitoring and Safety Committee (IDMC). A determination by the IDMC that there is a potential harm to participants or the environment caused by the interventions will result in discontinuation of the study.

If an individual wants to terminate his/her participation, no further follow-up will be performed. In the case of a study child leaving during year 1, they will be replaced in year 2 of the study (May-June 2013). There will be no replacement during the surveillance period either year. If a school opts out of the study before July in either year, replacement by a neighbouring school will be considered.

### Ethical considerations

This study is conducted in accordance with the principles set forth in the International Conference on Harmonization Tripartite Guideline of Good Clinical Practice and the Declaration of Helsinki. The study was approved by the Thai Ethics Review Board. Using a crossover, school-randomised design means that every individual will be equally exposed to the risks of dengue infection and to possible benefits of the intervention, taken over the entire trail period. Serious adverse effects of wearing the impregnated clothes will be reported to the Ethics Review Board within 24 h.

## Discussion

Reducing the incidence of dengue in school children not only reduces morbidity and mortality, but also reduces the number of school days lost, increases school performance and reduces the economic burden on parents. If our study proves that impregnated school uniforms reduce dengue infections in children, this would be a novel, simple and cost-effective intervention that is feasible, safe and scalable in resource-limited countries. Moreover, it should be community-based. However, if the null hypothesis is not rejected, then we have to elaborate on reasons why the intervention did not work. Potential reasons could be that the time an impregnated school uniform is worn during the day is not sufficiently long to protect against mosquito bites. Furthermore, the exposure to mosquito bites could possibly be higher during the daytime after school activities. Lastly, the waning of the knockdown effect of impregnated uniforms may be enhanced through washing and drying techniques in tropical countries that are different to those used in Western countries.

## Conclusion

This is a community effectiveness trial. Although a significant knockdown and repellence effect of impregnated clothing has been reported under ‘ideal’ conditions (efficacy), this needs to be tested in ‘real life’ under field conditions (effectiveness). There will be many confounding factors that will have an impact on the risk of transmission, for example, school uniforms typically do not cover the full body, they are not worn all day long, they are usually not worn during weekends and holidays, and lastly, the washing conditions in tropical countries (frequency of washing, drying in the sun, aggressive detergents, etc.) may expedite the waning of the knockdown effect over time.

## Trial status

The trial and enrolment at the time of submission.

## Competing interests

The authors declare that they have no competing interests.

## Authors’ contributions

AWS had the study idea and conceptualised the trial. AWS, PB and PK designed the trial. PB did the sample size calculations. PK, PO, PM and LS are responsible for the trial implementation. AWS wrote the paper. VL, SWL, PB, JL, PK, DG, YT and SB contributed to the writing of the paper. All authors read and approved the final manuscript.
